# Spleen tyrosine kinase/FMS-like tyrosine kinase-3 inhibition in relapsed/refractory B-cell lymphoma, including diffuse large B-cell lymphoma: updated data with mivavotinib (TAK-659/CB-659)

**DOI:** 10.18632/oncotarget.28352

**Published:** 2023-01-26

**Authors:** Leo I. Gordon, Reem Karmali, Jason B. Kaplan, Rakesh Popat, Howard A. Burris, Silvia Ferrari, Sumit Madan, Manish R. Patel, Giuseppe Gritti, Dima El-Sharkawi, F. Ian Chau, John Radford, Jaime Pérez de Oteyza, Pier Luigi Zinzani, Swaminathan P. Iyer, William Townsend, Harry Miao, Igor Proscurshim, Shining Wang, Shilpi Katyayan, Ying Yuan, Jiaxi Zhu, Kate Stumpo, Yaping Shou, Cecilia Carpio, Francesc Bosch

**Affiliations:** ^1^Division of Hematology and Oncology, Northwestern University Feinberg School of Medicine and the Robert H. Lurie Comprehensive Cancer Center, Chicago, IL 60611, USA; ^2^Department of Haematology, NIHR/UCLH Clinical Research Facility, University College London Hospitals NHS Foundation Trust, London, UK; ^3^Drug Development, Sarah Cannon Research Institute/Tennessee Oncology, Nashville, TN 37203, USA; ^4^Dipartimento di Oncologia ed Ematologia, Ospedale Papa Giovanni XXIII, Bergamo, Italy; ^5^Division of Hematology and Oncology, Cancer Therapy and Research Center at University of Texas Health Science Center, San Antonio, TX 78229, USA; ^6^Drug Development Unit, Florida Cancer Specialists/Sarah Cannon Research Institute, Sarasota, FL 34232, USA; ^7^Department of Medicine, Royal Marsden Hospital, Sutton, Surrey, UK; ^8^NIHR Clinical Research Facility, The Christie NHS Foundation Trust and University of Manchester, Manchester Academic Health Science Centre, Manchester, UK; ^9^Hematology, Hospital Universitario HM Sanchinarro, Madrid, Spain; ^10^IRCCS Azienda Ospedaliero-Universitaria di Bologna, Istituto di Ematologia “Seràgnoli”, Bologna, Italy; ^11^Dipartimento di Medicina Specialistica, Diagnostica e Sperimentale, Università di Bologna, Bologna, Italy; ^12^Department of Hematology and Oncology, Houston Methodist Cancer Center, Houston, TX 77030, USA; ^13^Oncology Clinical Science, Takeda Development Center Americas, Inc. (TDCA), Lexington, MA 02421, USA; ^14^Servei d’Hematologia, Vall d’Hebron Hospital Universitari, Experimental Hematology, Vall d’Hebron Institute of Oncology (VHIO), Vall d’Hebron Barcelona Hospital Campus, Barcelona, Spain; ^15^Departament de Medicina, Universitat Autònoma de Barcelona, Bellaterra, Spain; ^16^Current affiliation: Division of Hematology and Oncology, Banner MD Anderson Cancer Center, Gilbert, AZ 85234, USA; ^17^Current affiliation: Department of Haematology, Royal Marsden Hospital, Sutton, Surrey, UK; ^18^Current affiliation: Department of Lymphoma and Myeloma, University of Texas MD Anderson Cancer Center, Houston, TX 77030, USA; ^19^Current affiliation: Biostatistics, Labcorp Drug Development, Princeton, NJ 08540, USA

**Keywords:** Non-Hodgkin’s lymphoma, DLBCL, relapsed/refractory, SYK inhibitor, TAK-659

## Abstract

We report an updated analysis from a phase I study of the spleen tyrosine kinase (SYK) and FMS-like tyrosine kinase 3 inhibitor mivavotinib, presenting data for the overall cohort of lymphoma patients, and the subgroup of patients with diffuse large B-cell lymphoma (DLBCL; including an expanded cohort not included in the initial report).

Patients with relapsed/refractory lymphoma for which no standard treatment was available received mivavotinib 60–120 mg once daily in 28-day cycles until disease progression/unacceptable toxicity.

A total of 124 patients with lymphoma, including 89 with DLBCL, were enrolled. Overall response rates (ORR) in response-evaluable patients were 45% (43/95) and 38% (26/69), respectively. Median duration of response was 28.1 months overall and not reached in DLBCL responders. In subgroups with DLBCL of germinal center B-cell (GCB) and non-GCB origin, ORR was 28% (11/40) and 58% (7/12), respectively. Median progression free survival was 2.0 and 1.6 months in the lymphoma and DLBCL cohorts, respectively. Grade ≥3 treatment-emergent adverse events occurred in 96% of all lymphoma patients, many of which were limited to asymptomatic laboratory abnormalities; the most common were increased amylase (29%), neutropenia (27%), and hypophosphatemia (26%).

These findings support SYK as a potential therapeutic target for the treatment of patients with B-cell lymphomas, including DLBCL.

Trial registration: ClinicalTrials.gov number: NCT02000934.

## INTRODUCTION

Diffuse large B-cell lymphoma (DLBCL) is an aggressive histologic subtype of non-Hodgkin’s lymphoma and is the most common adult lymphoid malignancy in the Western world [1–4]. Although first-line R-CHOP treatment (rituximab plus cyclophosphamide, doxorubicin, vincristine, and prednisone) is curative for an estimated 60% of patients, outcomes are poor for those who are refractory to initial treatment or who relapse [4–9].

Multi-agent salvage chemotherapy followed by autologous stem-cell transplant (ASCT) has been the standard treatment for relapsed or refractory DLBCL [8, 10–12]. However, many patients are either ineligible for this intensive approach or do not respond adequately. More recently, several novel salvage therapeutic approaches, including cellular therapies, antibody drug conjugates, and bi-specific antibodies have been developed [13–17]. However, some of these novel therapies, particularly CD19-targeted chimeric antigen receptor (CAR) T-cell therapy, are associated with significant toxicities [14], and therefore additional novel options are needed.

Mivavotinib (TAK-659/CB-659) is an investigational, oral, reversible, potent dual inhibitor of spleen tyrosine kinase (SYK) and FMS-like tyrosine kinase 3 (FLT3) [18]. SYK is an essential component of the B-cell receptor signaling pathway; abnormal SYK signaling has been implicated in the pathogenesis of DLBCL and several other B-cell malignancies [18–22].

The safety, tolerability, and preliminary efficacy of mivavotinib were investigated in a phase I first-in-human dose escalation and expansion study conducted in patients with relapsed or refractory solid tumors or B-cell lymphomas, including DLBCL [23]. The maximum tolerated dose (MTD) was determined to be 100 mg once daily (QD) and anti-tumor activity was observed at doses of 60 mg to 120 mg QD. The most frequently occurring treatment-emergent adverse events (TEAE) were isolated aspartate aminotransferase (AST) elevations, pyrexia, and increased amylase, although abnormalities in clinical laboratory parameters were generally not associated with symptoms and were reversible upon treatment discontinuation.

Mivavotinib demonstrated anti-tumor activity in patients with relapsed or refractory B-cell lymphomas across different histological subtypes, including DLBCL [23]. The overall response rates (ORR) across all B-cell lymphoma subtypes in the intention-to-treat (ITT) population ranged from 20–50%. In patients with DLBCL (*n* = 53) the ORR was 23% in the ITT population (28% in the response-evaluable population), with a high proportion of those patients (19% of the response-evaluable population) achieving a complete response (CR). At the data cut-off (April 2018), the median treatment duration in patients with DLBCL was 14.3 months. The median duration of response (DOR) was not estimable (NE) due to ongoing responses in several patients. Based on these data, further evaluation was warranted, particularly in the DLBCL cohort [23].

The current analysis provides updated results for these lymphoma patients with extended follow-up. Also included are data for an additional cohort of patients with DLBCL (*n* = 36) who were enrolled to expand testing of mivavotinib safety and efficacy prior to the opening of a planned phase II study (NCT03123393) [24]. Data from these patients were not included in the initial analysis. Here, we report results for the complete DLBCL cohort (*n* = 89), including data from both the original and additional DLBCL escalation and expansion cohorts, and updated data for all lymphoma patients in this first-in-human study.

## RESULTS

### Patients

A total of 124 patients were enrolled; 17 patients were enrolled in the dose escalation phase and received mivavotinib at the following doses: 60 mg QD (*n* = 4), 80 mg QD (*n* = 3), 100 mg QD (*n* = 11), and 120 mg QD (*n* = 1); 107 patients were enrolled in the expansion phase and received 100 mg QD. Overall, 89 patients had DLBCL (72%), 23 had indolent non-Hodgkins lymphoma (iNHL) (19%), 6 had chronic lymphocytic leukemia (CLL) (5%), 5 had mantle cell lymphoma (MCL) (4%), and 1 patient had Epstein Barr virus-positive post-transplant lymphoproliferative disorder (EBV+PTLD) (1%). The iNHL subgroup included 16 patients with follicular lymphoma, 2 patients with mucosa-associated lymphoid tissue lymphoma, 2 patients with nodal marginal zone B-cell lymphoma, 1 patient with B-cell lymphoplasmacytic lymphoma/immunocytoma, 1 patient with B-cell small lymphocytic lymphoma and 1 patient with splenic marginal zone lymphoma.

**Figure 1:**
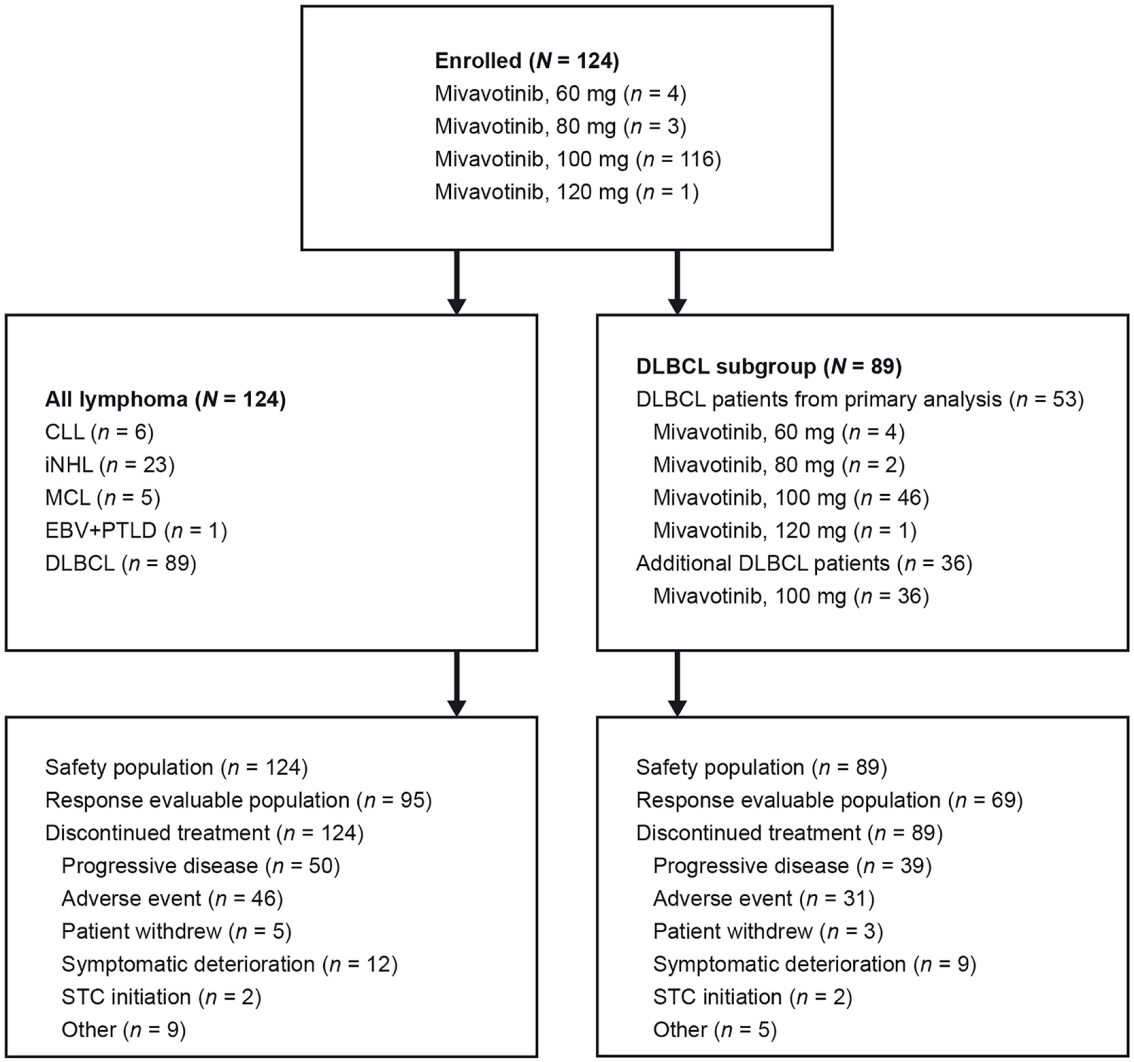
Patient disposition flow diagram.

Of the patients with DLBCL, 12 were enrolled during dose escalation, 41 were enrolled to the first expansion cohort, and 36 were enrolled to the second expansion cohort. By data cut-off, treatment had been discontinued in all 124 patients. Patient disposition is summarized in Figure 1.

Patient baseline demographics and disease characteristics for all lymphoma patients and all DLBCL patients are shown in Table 1. The median age, both in all patients with lymphoma and within the DLBCL subgroup, was 66 years (range 23–91). The median time since diagnosis was 21.8 months (range 0.3–269.3) for all patients with lymphoma and 16.4 months (range 0.3–269.3) for patients with DLBCL. Eighty-one patients with lymphoma (65%), including 60 patients with DLBCL (67%), were Ann Arbor stage III–IV at diagnosis, with 21% and 16%, respectively, having evidence of bone marrow involvement at study entry. Disease was classified as germinal center B-cell (GCB) in 47 patients, non-GCB in 18 patients, and unknown origin in 24 patients. The median number of prior lines of therapy was 3 (range 1–9) in all patients with lymphoma and within the DLBCL subgroup, with 17% and 15%, respectively, having previously undergone ASCT. Disease characteristics for DLBCL patients by dose escalation and expansion cohort are shown in Supplementary Table 1. The baseline characteristics were generally similar between the two DLBCL expansion cohorts, with the exception of median time since diagnosis, which was slightly shorter in the second expansion cohort.

**Table 1:** Baseline demographic and disease characteristics

	All DLBCL *n* = 89	All lymphomas^a,b^ *N* = 124
Age (years), median (range)	66 (23–91)	66 (23–91)
Gender (%), male/female	62/38	61/39
Race (%), white/other	97/2^c^	96/3^c^
Disease characteristics		
Time since diagnosis (months), median (range)	16.4 (0.3–269.3)	21.8 (0.3–269.3)
Tumor node metastases/Ann Arbor stage at diagnosis, *n* (%)	60 (67)	81 (65)
I	3 (3)	4 (3)
II	11 (12)	13 (10)
III	19 (21)	22 (18)
IV	41 (46)	59 (48)
Bone marrow involvement at entry, *n* (%)	14 (16)	26 (21)
Molecular/genetic classification (DLBCL only), *n* (%)		
GCB/non-GCB	47 (72^d^)/18 (28^d^)	
Double/triple hit	11 (16^e^)	
Treatment history		
Prior lines of therapy, median (range)	3 (1–9)	3 (1–9)
Prior autologous transplant, *n* (%)	13 (15)	21 (17)

^a^DLBCL (*n* = 89), iNHL (*n* = 23), CLL (*n* = 6), MCL (*n* = 5), EBV+PTLD (*n* = 1); ^b^Includes 36 additional patients with DLBCL and 2 additional patients with iNHL in addition to those included in the previously published analysis; ^c^Race not reported for 1 patient; ^d^Percentage of *n* = 65 patients with known molecular classification (*n* = 24 patients had unknown classification); ^e^Percentage of *n* = 67 patients in whom genetic classification was assessed.

### Efficacy

**Figure 2:**
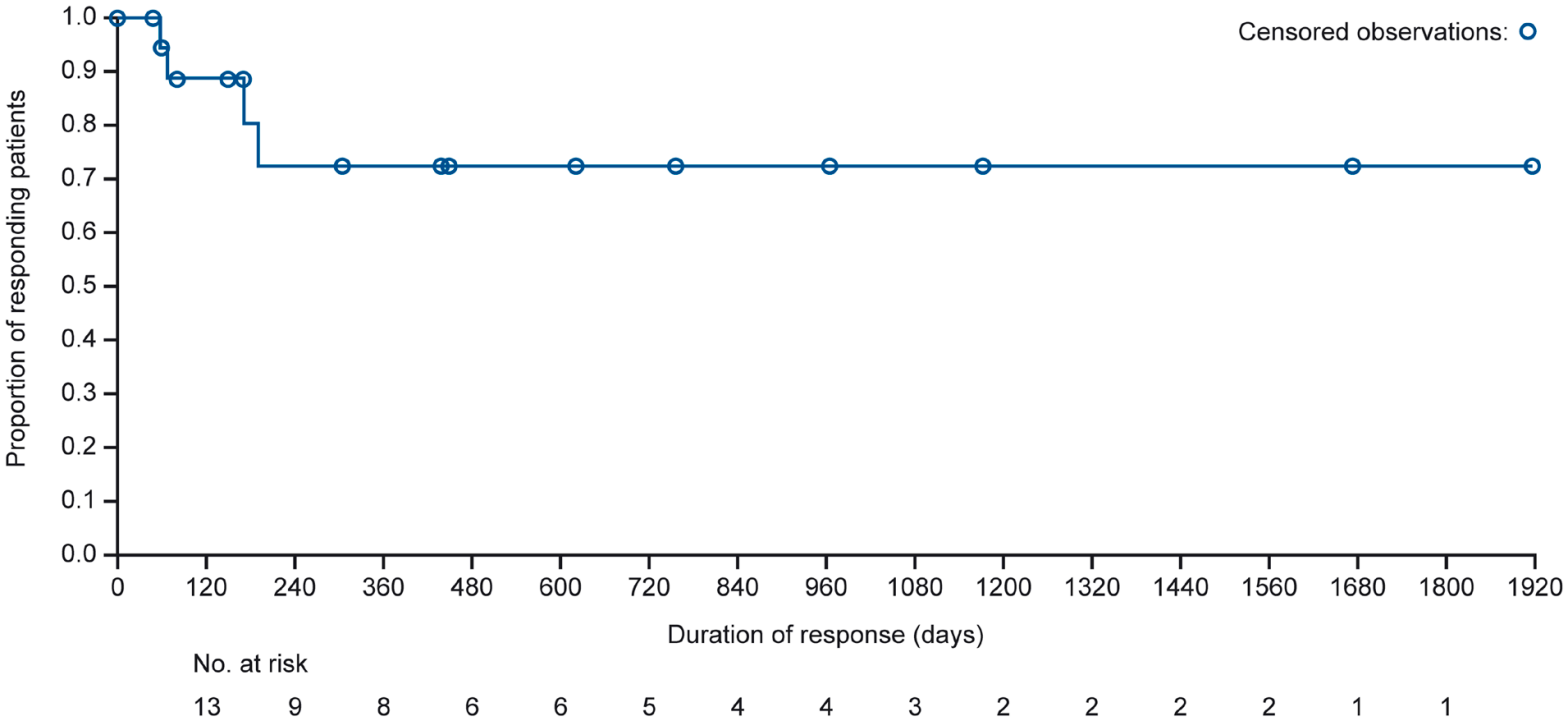
Kaplan–Meier curve for estimated median DOR in the DLBCL combined cohort (response-evaluable population). DOR was defined as the time from the date of first documentation of PR or better to the date of first documentation of PD or relapse. Among patients with CR, no patients had a PD/relapse event, and data were censored with a range of less than one month (1 day) to 63.0 months with responses ongoing for all 14 patients at the time of data cut. Among patients with PR, 4 patients had a PD/relapse event, and data were censored for the remaining 8 patients.

Best overall responses to mivavotinib are shown in Table 2. A total of 95 patients with lymphoma (77%) were evaluable for response; 19 patients (20%) achieved a CR and 24 (25%) achieved a partial response (PR), resulting in an ORR of 45% (95% confidence interval [CI], 35.0–55.8); 40 (42%) patients had a best response of progressive disease (PD). Median time to response among all lymphoma patients was 1.8 months. The median DOR in patients with lymphoma achieving CR or PR was 28.1 months (95% CI, 5.8–NE). Within the DLBCL subgroup, 69 patients (78%) were response-evaluable; of these, 14 patients (20%) achieved CR and 12 (17%) achieved PR, resulting in an ORR of 38% (95% CI, 26.3–50.2). Thirty-five (51%) patients with DLBCL had a best response of PD. In responding patients with DLBCL (*n* = 26), the median DOR was NE (95% CI, 6.3 months – NE) (Figure 2), 8 patients (31%) had responses lasting >12 months and 5 patients (19%) had responses lasting >24 months. Among DLBCL patients with PR (*n* = 12), the median DOR was 5.7 months (95% CI, 1.9–NE); among patients with CR, no patients had a PD/relapse event, and data were censored with a range of less than one month (1 day) to 63.0 months with responses ongoing for all 14 patients at the time of data cut.

**Table 2:** Best overall response

Response, *n* (%)	DLBCL escalation	DLBCL cohort 1	DLBCL cohort 2	DLBCL cohorts (combined)	All lymphomas
ITT population, *n*	12	41	36	89	124
ORR (CR + PR)	4 (33)	8 (20)	14 (39)	26 (29)	43 (35)
Response-evaluable population, *n*	11	33	25	69	95
ORR (CR + PR)	4 (36)	8 (24)	14 (56)	26 (38)	43 (45)
95% CI	10.9–69.2	11.1–42.3	34.9–75.6	26.3–50.2	35.0–55.8
Clinical benefit (CR + PR + SD)	6 (55)	14 (42)	14 (56)	34 (49)	55 (58)
CR	3 (27)	6 (18)	5 (20)	14 (20)	19 (20)
PR	1 (9)	2 (6)	9 (36)	12 (17)	24 (25)
SD	2 (18)	6 (18)	0	8 (12)	12 (13)
PD	5 (45)	19 (58)	11 (44)	35 (51)	40 (42)

Percentages are based on the total number of patients in the response-evaluable population in each column. 2-sided 95% exact binomial CIs were used. Abbreviation: SD: stable disease.

Within the subgroup of patients with DLBCL (excluding those enrolled in dose escalation), 33 patients in the first expansion cohort were evaluable for response and 25 in the second cohort were evaluable for response. The ORR was 24% (95% CI, 11.1–42.3) in the first DLBCL cohort (18% with CR and 6% with PR) and 56% (95% CI, 34.9–75.6) in the second DLBCL cohort (20% with CR and 36% with PR). Considering all response-evaluable patients with DLBCL enrolled in the study (*n* = 69), 40 patients had GCB type DLBCL with an ORR of 28% (including 23% with CR), and 12 patients had non-GCB type DLBCL with an ORR of 58% (8% with CR); 17 patients had unknown GCB type (ORR 47%; CR rate 24%) (Supplementary Table 2). Responses were maintained at data cut-off/last follow up in all responding patients with GCB type DLBCL (*n* = 11) and in 6/7 responding patients with non-GCB DLBCL; DOR was therefore NE. In the subgroup with unknown cell of origin, median DOR was 6.3 months (95% CI, 1.9–NE). The ORR among DLBCL patients with 1 vs. >1 prior lines of therapy was 50% vs. 35%, while the ORR among DLBCL patients with prior transplant vs. without prior transplant was 33% vs. 39%.

**Figure 3:**
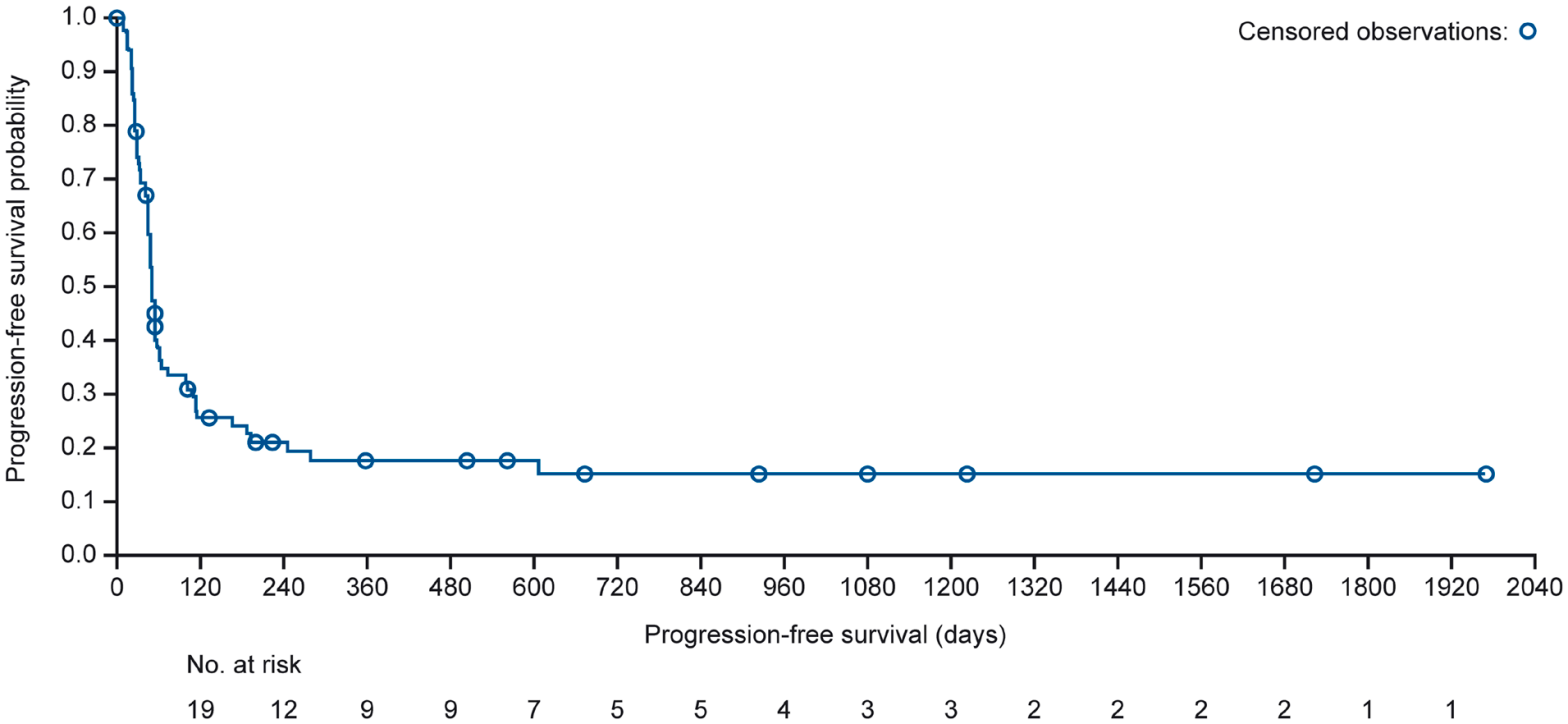
Kaplan–Meier curve for estimated median PFS in the DLBCL combined cohort (safety population). PFS is defined as the time from the date of the first study treatment administration to the date of the first documentation of progressive disease or death.

The median progression-free survival (PFS) in all patients with lymphoma was 2.0 months (95% CI, 1.6–3.3); 86 patients (69%) experienced PFS events, of which 53 (62%) were due to progression and 33 (38%) were due to death. In patients with DLBCL, the median PFS was 1.6 months (95% CI, 1.5–1.9), and 67 patients (75%) experienced PFS events; 41 (61%) were due to disease progression and 26 (39%) were due to death (Figure 3). Median time to progression (TTP) was 3.7 months (95% CI, 1.9–7.7) in the overall lymphoma group and 1.8 months (95% CI, 1.6–3.7) in the DLBCL subgroup.

The median overall survival (OS) was 8.3 months (95% CI, 3.7–NE) in patients with lymphoma and 3.9 months (95% CI, 2.1–NE) in patients with DLBCL. Overall, 52 patients with lymphoma (42%), including 42 patients in the DLBCL cohorts (47%), died within the time period between the first dose and the last follow-up.

Within the DLBCL response-evaluable population (*n* = 69), median PFS was 19.9 months (95% CI, 6.3–NE) in responding patients (*n* = 26 patients with CR or PR), compared with 1.5 months (95% CI, 1.0–1.6) in non-responders (*n* = 43). Median OS was not reached in responders (95% CI, 8.3–NE) and 3.3 months (95% CI, 2.1–3.9) in non-responders. Considering patient subgroups based on cell of origin, median PFS was similar in patients with GCB (1.6 months [95% CI, 1.3–2.1]), non-GCB (1.7 months [1.1–3.7]) and unknown origin (1.6 months [0.9–3.5]) DLBCL (Supplementary Figure 1A). However, there was a higher proportion of PD events in GCB patients, while there were more deaths in non-GCB patients, and OS differed accordingly, with median OS 19.9 months (95% CI, 2.4–NE) and 2.1 months (95% CI, 1.5–NE), respectively. Median OS in the unknown origin subgroup was 3.7 months (95% CI, 1.2–NE) (Supplementary Figure 1B).

### Safety

All patients received at least one dose of mivavotinib and were evaluable for safety. Patients received a median of 2 treatment cycles (range 1–68), and the median treatment duration was 6.8 weeks for all patients with lymphoma and 6.0 weeks for patients with DLBCL. Safety is summarized in Tables 3–5 for the total patients with lymphoma and for the subgroup of patients with DLBCL. Safety was generally consistent between the overall lymphoma population and the DLBCL subgroup and so is only described for patients with lymphoma.

**Table 3:** Overview of TEAEs (safety population)

Adverse event *n*, (%)	DLBCL cohorts (combined) *n* = 89	All lymphomas *N* = 124
Any TEAEs	89 (100)	124 (100)
Related	82 (92)	114 (92)
Not related	88 (99)	122 (98)
Grade 1	85 (96)	117 (94)
Grade 2	83 (93)	112 (90)
Grade ≥3	85 (96)	119 (96)
Grade ≥3 related	65 (73)	94 (76)
Leading to discontinuation	32 (36)	48 (39)
Serious TEAEs	65 (73)	94 (76)
Related	15 (17)	33 (27)
Not related	58 (65)	79 (64)
Leading to discontinuation	20 (22)	32 (26)
Deaths	32 (36)	39 (31)
Related	2	4

TEAEs are defined as any adverse event that occurs after administration of the first dose of study treatment through 28 days after last dose of study treatment, or until the start of subsequent antineoplastic therapy. Deaths occurred within 28 days of last treatment dose. Percentages are based on the number of patients in each treatment group for the designated population of this table.

**Table 4:** Most frequent TEAEs occurring in ≥10% of patients by preferred term (safety population)

Preferred term (*n* [%])	DLBCL cohorts (combined) *n* = 89	All lymphomas *N* = 124
Any TEAE	89 (100)	122 (98)
AST increased	56 (63)	75 (60)
Pyrexia	44 (49)	69 (56)
Amylase increased	43 (48)	57 (46)
Hypophosphatemia	34 (38)	51 (41)
Anemia	35 (39)	50 (40)
Blood CPK increased	37 (42)	50 (40)
Diarrhea	33 (37)	48 (39)
Lipase increased	34 (38)	45 (36)
Fatigue	30 (34)	43 (35)
ALT increased	32 (36)	39 (31)
Neutropenia	29 (33)	39 (31)
Nausea	25 (28)	37 (30)
Cough	22 (25)	34 (27)
Thrombocytopenia	24 (27)	33 (27)
Asthenia	22 (25)	32 (26)
Decreased appetite	20 (22)	28 (23)
Periorbital edema	17 (19)	27 (22)
Blood alkaline phosphate increased	19 (21)	26 (21)
Constipation	19 (21)	26 (21)
Vomiting	17 (19)	26 (21)
Pneumonia	14 (16)	25 (20)
Edema peripheral	17 (19)	24 (19)
Hypokalemia	14 (16)	23 (19)
Abdominal pain	18 (20)	22 (18)
Headache	11 (12)	22 (18)
Blood creatine increased	16 (18)	20 (16)
Dyspnea	14 (16)	19 (15)
Cytomegalovirus infection reactivation	14 (16)	18 (15)
Urinary tract infection	10 (11)	17 (14)
Blood lactate dehydrogenase increased	12 (13)	16 (13)
Chills	8 (9)	16 (13)
Gamma-glutamyltransferase increased	10 (11)	16 (13)
Hypertension	12 (13)	16 (13)
Stomatitis	10 (11)	16 (13)
Back pain	10 (11)	15 (12)
Night sweats	9 (10)	14 (11)
Oral candidiasis	6 (7)	14 (11)
Hyponatremia	8 (9)	13 (10)
Rash maculo-papular	10 (11)	13 (10)

TEAEs are defined as any adverse event that occurs after administration of the first dose of study treatment through 28 days after the last dose of study treatment, or until the start of subsequent antineoplastic therapy. Abbreviations: ALT: alanine aminotransferase; CPK: creatine phosphokinase.

**Table 5:** Most common grade ≥3 TEAEs occurring in ≥10% of patients by preferred term (safety population)

Preferred term, *n* (%)	DLBCL cohorts (combined) *n* = 89	All lymphomas *N* = 124
Patients with grade ≥3 TEAE	85 (96)	119 (96)
Increased amylase	28 (31)	36 (29)
Neutropenia	26 (29)	34 (27)
Hypophosphatemia	19 (21)	32 (26)
Anemia	15 (17)	23 (19)
Blood CPK increased	15 (17)	22 (18)
Lipase increased	15 (17)	22 (18)
Thrombocytopenia	13 (15)	19 (15)
Pneumonia	7 (8)	14 (11)
Pyrexia	8 (9)	13 (10)
AST increased	11 (12)	12 (10)

TEAEs are defined as any adverse event that occurs after administration of the first dose of study treatment through 28 days after the last dose of study treatment, or until the start of subsequent antineoplastic therapy.

All patients with lymphoma experienced at least one TEAE; 96% experienced a grade ≥3 TEAE, and 76% experienced a grade ≥3 TEAE considered by the investigator to be related to mivavotinib (Table 3). The most common TEAEs occurring in ≥10% of all patients were increased AST (60%), pyrexia (56%), and increased amylase (46%) (Table 4). The most frequent grade ≥3 TEAEs occurring in ≥10% of all patients were increased amylase (29%), neutropenia (27%), and hypophosphatemia (26%) (Table 5). Granulocyte colony stimulating factor (G-CSF) was administered to 28 patients for the management of grade ≥3 neutropenia. Overall, 39% of patients with lymphoma experienced TEAEs resulting in mivavotinib discontinuation; the most common TEAEs leading to discontinuation were pneumonia (*n* = 6), pneumonitis (*n* = 3), respiratory failure (*n* = 4), and neutropenia (*n* = 4). Serious TEAEs were experienced by 76% of patients; 27% of patients had serious TEAEs which were related to mivavotinib, and 26% had serious TEAEs which resulted in discontinuation. Overall, 39 patients with lymphoma died on study; 4 deaths were considered related to mivavotinib and were due to complications from pneumocystis pneumonia, multiorgan failure, respiratory failure and disseminated varicella (*n* = 1 each).

## DISCUSSION

Primary data from this phase I, first-in-human study investigating the safety, tolerability, and preliminary efficacy of SYK/FLT3 inhibitor mivavotinib, conducted in patients with advanced solid tumors or lymphoma malignancies, were previously reported [23]. Here we provide updated results for the total cohort of patients with lymphoma (*N* = 124) and present findings for an expanded subgroup of patients with DLBCL (*n* = 89). In this updated analysis, mivavotinib demonstrated encouraging efficacy in patients with lymphoma and in the subgroup of patients with DLBCL, with ORRs of 45% and 38%, respectively, and a CR rate of 20% in both groups. The safety profile was consistent with the previous report, with no new signals identified.

The ORR and CR rates reported here for all patients with lymphoma including those with DLBCL are clinically meaningful, given the limited treatment options and poor outcomes for these patients with relapsed or refractory disease [1–4]. In addition, some of the patients were heavily pretreated with a median of 3 prior lines of therapy. Responses were also slightly improved in the second DLBCL expansion cohort (56%) vs. the first expansion cohort (24%), which might be due to differences in eligibility criteria (see Methods section) between the two cohorts. There was also a higher proportion of patients with non-GCB lymphoma in the second cohort (36% vs. 21%; Supplementary Table 1). However, baseline disease characteristics were generally consistent between the two cohorts, except for a shorter median time since diagnosis in the second. Similarly, the ORR was higher in patients with only 1 prior line of therapy (50%) vs. patients with >1 prior line of therapy (35%). Further research would be required to determine whether this reflects a higher benefit of mivavotinib therapy earlier in the treatment paradigm.

Meaningful response rates were observed in both GCB and non-GCB DLBCL subtypes, consistent with the findings of our initial report [23]. There are known biological differences between these two DLBCL subtypes, as activated B-cell-like DLBCL (which comprises the majority of non-GCB) is known to be more dependent on B-cell receptor (BCR) pathway signaling involving SYK than GCB DLBCL [2, 4, 25, 26]. Although the patient numbers were small (*n* = 12 patients with non-GCB subtype), there was a higher response rate observed in patients with non-GCB DLBCL (58% vs. 28%), which might have been a result of these biological differences; however, the majority were partial responses which did not translate into longer OS. In contrast, patients with GCB subtype had a higher rate of CR (23% vs. 8%) and longer OS (19.9 vs. 2.1 months), which might have been expected since some studies have reported a more aggressive course with non-GCB DLBCL subtypes resulting in worse outcomes for patients [27]. Given the differences in eligibility criteria and the known heterogeneity of DLBCL pathogenesis and presentation, further genomic analyses of non-GCB and GCB DLBCL subtypes to characterize markers for response to targeted treatment could enable the identification of patient populations and DLBCL subtype(s) which may derive therapeutic benefit from a given treatment. For example, mutations in genes for key regulators of the B-cell and/or Toll-like receptor pathways such as MYD88 or CD79B [28] could suggest potential for modification by treatments targeting these pathways, including SYK/FLT3 inhibitors such as mivavotinib.

Although population-level outcomes data are limited for patients with relapsed or refractory DLBCL, in the large retrospective SCHOLAR study of 636 patients, estimated objective response rates were 26% with a CR rate of 7% and a median OS of 6.3 months [29]. The response rates reported in the present study (38% ORR, including 20% CR) are improved compared with those estimated in this retrospective study, despite the median OS of 3.9 months being lower than what might be expected in this patient population. In addition, agents which recently received US Food and Drug Administration accelerated approval for treatment of DLBCL based on encouraging response rates such as selinexor [30], polatuzumab vedotin (in several combinations), tafasitamab (in combination with lenalidomide), and CAR T-cell therapy such as axicabtagene ciloleucel and tisagenlecleucel [14, 31], have achieved improved, or similar response rates than those reported with mivavotinib. However, a subsequent phase II study of mivavotinib monotherapy, which enrolled 49 patients with relapsed or refractory DLBCL (with similar eligibility criteria to the second expansion cohort) was terminated due to lack of efficacy [24]. Given the smaller number of patients in the study and perhaps because not all patients started dosing at 100 mg QD, with 25 patients starting treatments at 60 mg to evaluate a ramp up dosing schedule, it is possible that such differences contributed to the lack of efficacy observed. Altogether, these data suggest that a biomarker selection strategy, further dose refinement, and combinations of mivavotinib with other agents in DLBCL warrant further exploration. An ongoing phase I study is investigating the safety and efficacy of mivavotinib with R-CHOP as first-line treatment for patients with high-risk DLBCL [32], while a phase II study of mivavotinib in patients with relapsed or refractory non-GCB DLBCL is investigating efficacy in subgroups defined according to genetic biomarkers [33].

CAR T-cell therapy is expected to become the new standard of care for relapsed or refractory DLBCL [34, 35]. Several CD19-targeted CAR T-cell therapy agents have been approved for treatment, including axicabtagene ciloleucel [14], lisocabtagene maroleucel [36], and tisagenlecleucel [37, 38]. Considering these recent developments, there may be a role for mivavotinib combinations in the treatment pathway of patients with relapsed or refractory DLBCL, potentially as a bridge to CAR T-cell therapy or as an option post-CAR T-cell therapy.

Overall, safety findings were consistent with the primary analysis and toxicity was manageable despite a high proportion of patients experiencing grade ≥3 TEAEs (96% all cause, and 76% determined by the investigator as being related to mivavotinib). The most common grade ≥3 TEAEs were increased amylase, neutropenia and hypophosphatemia; other common grade ≥3 TEAEs included elevations in clinical laboratory investigations, including AST, amylase, lipase and blood creatine phosphokinase, which were largely asymptomatic and reversible upon dose reduction or discontinuation of the study drug, consistent with the initial analysis. The most common hematologic grade ≥3 TEAE was neutropenia (27%), with most of these patients receiving G-CSF support to manage it; this was largely expected due to the number of patients with bone marrow involvement at study entry. Other hematologic grade ≥3 TEAEs including anemia and thrombocytopenia occurred in 19% and 15% of patients, respectively. The most common metabolic disorder of grade ≥3 was hypophosphatemia (26%). Serious TEAEs were reported in 76% of patients; however, only 27% of patients had serious TEAEs determined by the investigator as being related to mivavotinib, and only 26% of patients had serious TEAEs that led to discontinuation. There were 39 on-study deaths among patients with lymphoma, 4 of which were considered related to mivavotinib and were due to complications from pneumocystis pneumonia, multiorgan failure, respiratory failure and disseminated varicella. The most common reasons for discontinuation were pneumonia and pneumonitis; further intervention to reduce the risk of opportunistic infection (e.g., *Pneumocystis jirovecii* pneumonia) or viral reactivation could further improve the safety profile of mivavotinib in future studies.

In conclusion, the anti-tumor efficacy of mivavotinib monotherapy observed in the primary analysis of this study was confirmed in our analysis of patients with lymphoma, including an expanded cohort of patients with relapsed or refractory DLBCL, with responses that were deep and durable. These findings support SYK as a potential therapeutic target for the treatment of this population of patients. Further investigation of markers to predict response to SYK inhibition, and research into possible mivavotinib treatment combinations, are needed to develop mivavotinib further, and to provide more extensive therapy options for patients with relapsed or refractory DLBCL who have limited treatment options.

## MATERIALS AND METHODS

### Study design

This was an open-label, multicenter, phase I, dose escalation and expansion study of QD, oral, single-agent mivavotinib in patients with advanced solid tumors or lymphoid malignancies. The full study design and methods have previously been reported [23]. Here we focus on aspects pertinent to patients with lymphoma, and particularly those with DLBCL.

Briefly, in the dose escalation phase, adults with a confirmed diagnosis of lymphoma for which no standard treatment was available were enrolled to receive escalating doses of oral mivavotinib (60 mg, 80 mg, 100 mg or 120 mg QD) in an accelerated 3+3 dose escalation design. In the expansion phase, patients with lymphoid malignancies received mivavotinib 100 mg QD (the MTD from the dose escalation phase) in one of six disease-specific cohorts: CLL, iNHL, MCL, EBV+PTLD and two separate DLBCL expansion cohorts. All patients in the escalation and expansion phases received oral mivavotinib at their assigned dosage in continuous 28-day cycles until disease progression or unacceptable toxicity.

### Patients

Lymphoma patients enrolled in both the escalation and expansion phases had histologically or cytologically confirmed lymphoma, according to the modified International Working Group (IWG) 2007 criteria for malignant lymphoma [39], or the International Workshop on Chronic Lymphocytic Leukemia 2008 criteria. Patients in the expansion cohorts also had at least one site of measurable or evaluable disease confirmed by computed tomography. Additional eligibility criteria included an Eastern Cooperative Oncology Group performance status of 0–1, adequate organ function, a life expectancy longer than 3 months, and recovery from reversible effects of prior anti-cancer therapy.

Patients enrolled in the first DLBCL expansion cohort had pathologically confirmed DLBCL with at least one site of measurable disease based on IWG criteria for malignant lymphoma [39], had relapsed or refractory disease after at least one line of therapy, and were ineligible for or had progressed after receiving high-dose chemotherapy/ASCT. Patients enrolled in the second DLBCL expansion cohort had histologically-confirmed DLBCL, including *de novo* DLBCL or transformed disease from iNHL, and had relapsed or refractory disease after ≥2 lines of chemotherapy (based on a standard of care which included rituximab plus anthracycline [or equivalent if contraindicated]) and an additional systemic chemotherapy as second-line salvage therapy (that may have included ASCT), but had not failed >4 prior lines of therapy. Patients enrolled in the second DLBCL expansion cohort could also have been previously treated with BCR in-pathway inhibitors not directly targeting SYK. For patients in both DLBCL cohorts, DLBCL cell of origin was determined by immunohistochemistry when available (local laboratory) and was classified as GCB or non-GCB [40]. Cytogenetic profiling was performed using fluorescence *in-situ* hybridization where available, although this was not mandated, and patients with multiple gene rearrangements (*MYC* and *BCL2* and/or *BCL6*) were identified as having double- or triple-hit lymphoma.

### Assessments

Efficacy endpoints including ORR, DOR, PFS, TTP and OS were analyzed for all patients with lymphoma, including the additional DLBCL expansion cohort, and for the full DLBCL subgroup (both expansion cohorts and the escalation cohort combined), based on data collected up to June 29, 2021. This report includes extended follow-up data for patients included in the initial analysis, as well as data for additional DLBCL patients. DOR was also analyzed among the separate DLBCL cohorts, and assessments of ORR were made in the GCB, non-GCB, or unknown GCB classified DLBCL subgroups.

Responses were assessed in patients who received at least one dose of study drug and had at least one post-baseline disease assessment (response-evaluable population). Assessments were performed at cycles 2, 4, 6, then every 3 cycles through cycle 24, and thereafter every 6 cycles (until disease progression or the start of alternative therapies). PFS, TTP and OS were evaluated in the ITT population, based on the time from the date of first study drug administration to the date of first documentation of PD (PFS/TTP) or death (PFS/OS).

The safety population was defined as all patients in any lymphoma cohort receiving at least one dose of study drug. Adverse events and toxicity were assessed continually during treatment and were graded in accordance with the National Cancer Institute Common Terminology Criteria for Adverse Events version 4.03.

Statistical analyses were primarily descriptive without formal hypothesis testing. Median DOR, PFS and OS were estimated using the Kaplan–Meier method.

### Data sharing statement

Requests for de-identified datasets for the results reported in this publication will be made available to qualified researchers following submission of a methodologically sound proposal. Data will be made available for such requests following online publication of this article and for 1 year thereafter in compliance with applicable privacy laws, data protection, and requirements for consent and anonymization. Calithera does not share identified participant data or a data dictionary.

## SUPPLEMENTARY MATERIALS



## Abbreviations

ALT: alanine aminotransferase
ASCT: autologous stem-cell transplant
AST: aspartate aminotransferase
BCR: B-cell receptor
CAR: CD19-targeted chimeric antigen receptor
CI: confidence interval
CLL: chronic lymphocytic leukemia
CPK: creatine phosphokinase
CR: complete response
DLBCL: diffuse large B-cell lymphoma
DOR: duration of response
EBV+PTLD: Epstein Barr Virus-positive post-transplant lymphoproliferative disorder
FLT3: FMS-like tyrosine kinase 3
GCB: germinal center B-cell
G-CSF: granulocyte colony stimulating factor
iNHL: indolent non-Hodgkin’s lymphoma
ITT: intention-to-treat
IWG: International Working Group
MCL: mantle cell lymphoma
MTD: maximum tolerated dose
NE: not estimable
ORR: overall response rate
OS: overall survival
PD: progressive disease
PFS: progression-free survival
PR: partial response
QD: once daily
R-CHOP: rituximab plus cyclophosphamide, doxorubicin, vincristine, and prednisone
SD: stable disease
SYK: spleen tyrosine kinase
TEAE: treatment-emergent adverse events
TTP: time to progression
