# Supplementary material for: Spleen tyrosine kinase/FMS-like tyrosine kinase-3 inhibition in relapsed/refractory B-cell lymphoma, including diffuse large B-cell lymphoma: updated data with mivavotinib (TAK-659/CB-659)
Source: Oncotarget. 2023 Jan 26;14:57–70. doi: 10.18632/oncotarget.28352 (PMC9882996; doi:10.18632/oncotarget.28352)
Supplement: Supplementary file 1 [file oncotarget-14-28352-s001.pdf]

Spleen tyrosine kinase/FMS-like tyrosine kinase-3 inhibition in relapsed/refractory B-cell lymphoma, including diffuse large B-cell lymphoma: updated data with mivavotinib (TAK-659/CB-659)

SUPPLEMENTARY MATERIALS

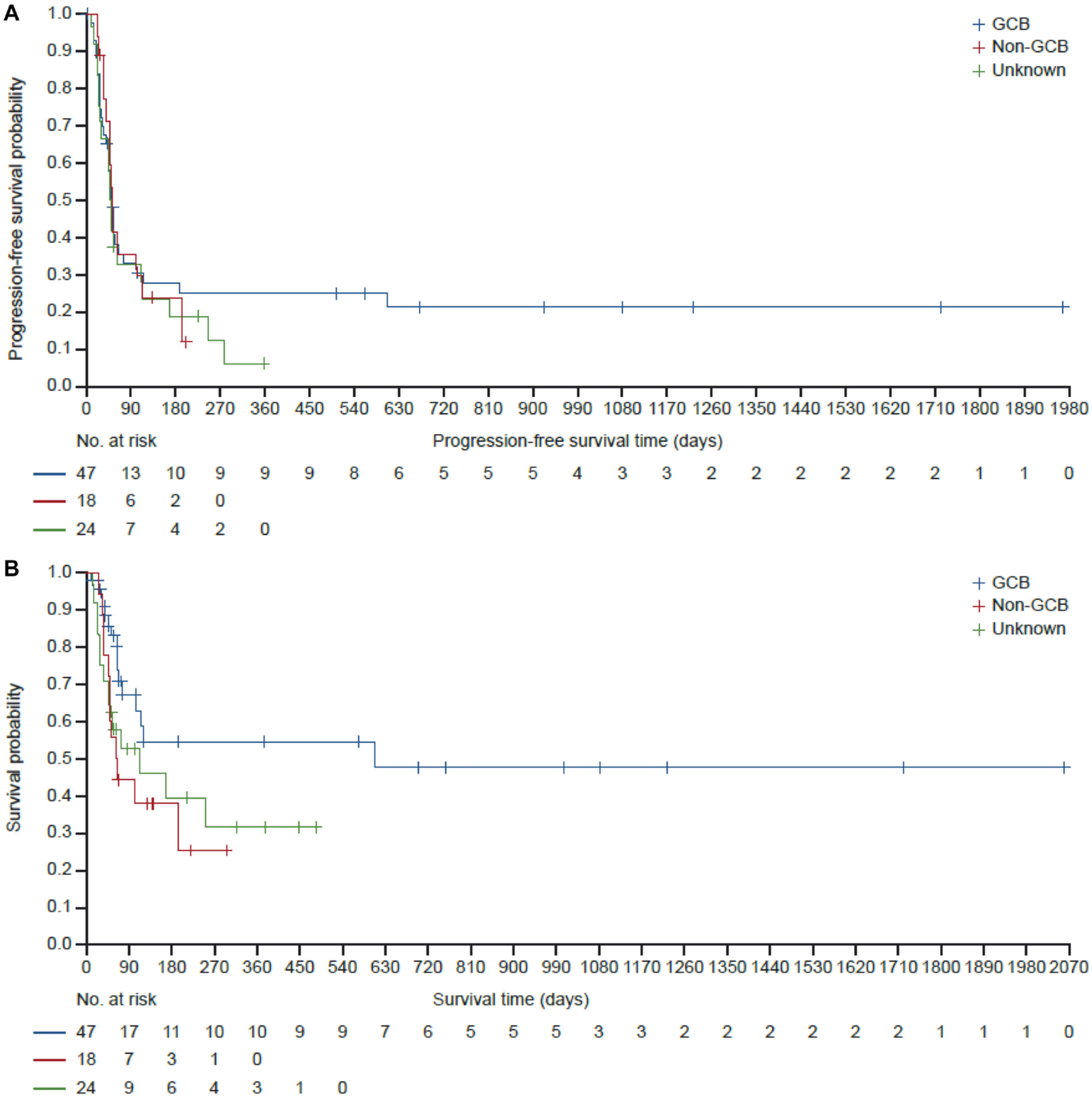

Supplementary Figure 1: PFS (**A**) and OS (**B**) in patients with GCB, non-GCB and unknown cell of origin DLBCL.

Supplementary Table 1: Baseline disease characteristics in DLBCL cohorts

	DLBCL escalation cohort *n* = 12	DLBCL expansion cohort 1 *n* = 41	DLBCL expansion cohort 2 *n* = 36	All DLBCL *n* = 89
Lymphoma disease type, *n* (%)				
Diffuse large B-cell lymphoma	1 (8)	41 (100)	36 (100)	78 (88)
Mature B-cell neoplasm	11 (92)	0	0	11 (12)
Disease variant type, *n* (%)				
De novo	6 (50)	25 (61)	25 (69)	56 (63)
Transformed	6 (50)	15 (37)	11 (31)	32 (36)
Unknown	0	1 (2)	0	1 (1)
Molecular classification, *n* (%)	*n = 12*	*n = 28*	*n = 25*	*n = 65*
GCB	9 (75)	22 (79)	16 (64)	47 (72)
Non-GCB	3 (25)	6 (21)	9 (36)	18 (28)
Unknown	0	13	11	24
Genetic classification, *n* (%)	*n = 11*	*n* = 26	*n* = 30	*n* = 67
Double hit	2 (18)	3 (12)	3 (10)	8 (12)
Triple hit	0	1 (4)	2 (7)	3 (4)
Not double hit or triple hit	9 (82)	22 (85)	25 (83)	56 (84)
Missing	1	15	6	22
Months since diagnosis, median (range)	32.8 (1.0–269.3)	17.3 (0.3–256.9)	13.6 (3.9–188.8)	16.4 (0.3–269.3)
ECOG performance status, *n* (%)				
0	8 (67)	9 (22)	8 (22)	25 (28)
1	4 (33)	32 (78)	25 (69)	61 (69)
2	0	0	3 (8)	3 (3)

Percentages are based on the number of patients in each cohort, except for molecular/genetic classification, where percentages are based on numbers shown with known status. Abbreviation: ECOG: Eastern Cooperative Oncology Group.

Supplementary Table 2: Best response by disease variant and cell of origin subgroup (response evaluable patients with DLBCL; *n* = 69)

DLBCL type	Evaluable	CR (%)	PR (%)	ORR (%)
DLBCL (all)	69	14 (20)	12 (17)	26 (38)
Disease variant				
De novo	40	6 (15)	7 (18)	13 (33)
GCB	19	4 (21)	2 (11)	6 (32)
Non-GCB	7	0	2 (29)	2 (29)
Unknown	14	2 (14)	3 (21)	5 (36)
Transformed	28	8 (29)	4 (14)	12 (43)
GCB	21	5 (24)	0	5 (24)
Non-GCB	4	1 (25)	3 (75)	4 (100)
Unknown	3	2 (67)	1 (33)	3 (100)
Unknown	1	0	1 (100)	1 (100)
Non-GCB	1	0	1 (100)	1 (100)

Cell of origin				
GCB[a]	40	9 (23)	2 (5)	11 (28)
Non-GCB[a]	12	1 (8)	6 (50)	7 (58)
Unknown[a]	17	4 (24)	4 (24)	8 (47)

[a]*De novo*, transformed and unknown DLCBL, combined.
